# Dose- and time-dependent effects of actomyosin inhibition on live mouse outflow resistance and aqueous drainage tissues

**DOI:** 10.1038/srep21492

**Published:** 2016-02-17

**Authors:** MinHee K. Ko, Eun Kyoung Kim, Jose M. Gonzalez, James C. Tan

**Affiliations:** 1 Doheny Eye Institute and Department of Ophthalmology, David Geffen School of Medicine at UCLA, Los Angeles, CA, USA

## Abstract

Actomyosin contractility modulates outflow resistance of the aqueous drainage tissues and intraocular pressure, a key pathogenic factor of glaucoma. We established methodology to reliably analyze the effect of latrunculin-B (Lat-B)-induced actin depolymerization on outflow physiology in live mice. A voltage-controlled microperfusion system for delivering drugs and simultaneously analyzing outflow resistance was tested in live C57BL/6 mice. Flow rate and perfusion pressure were reproducible within a coefficient of variation of 2%. Outflow facility for phosphate-buffered saline (0.0027 ± 0.00036 μL/min/mmHg; mean ± SD) and 0.02% ethanol perfusions (Lat-B vehicle; 0.0023 ± 0.0005 μL/min/mmHg) were similar and stable over 2 hours (p > 0.1 for change), indicating absence of a ‘washout’ artifact seen in larger mammals. Outflow resistance changed in graded fashion, decreasing dose- and time-dependently over 2 hours for Lat-B doses of 2.5 μM (p = 0.29), 5 μM (p = 0.039) and 10 μM (p = 0.001). Resulting outflow resistance was about 10 times lower with 10 μM Lat-B than vehicle control. The filamentous actin network was decreased and structurally altered in the ciliary muscle (46 ± 5.6%) and trabecular meshwork (37 ± 8.3%) of treated eyes relative to vehicle controls (p < 0.005; 5 μM Lat-B). Mouse actomyosin contractile mechanisms are important to modulating aqueous outflow resistance, mirroring mechanisms in primates. We describe approaches to reliably probe these mechanisms *in vivo*.

Aqueous drainage tissue actomyosin contractility^1^,^2^,^3^,^4^ modulates intraocular pressure (IOP) and aqueous humor outflow resistance, which becomes abnormal in glaucoma. Elucidating contractility-mediated mechanisms of outflow resistance is expected to provide important clues to glaucoma pathogenesis and therapeutic development, but currently there is a scarcity of accessible and validated live animal model platforms to support such studies.

The aqueous outflow system is complex in organization and function and important aspects of this complexity are lost when studies are performed *ex vivo* or *in vitro*. Traditionally, *in vivo* studies have been performed by anterior chamber perfusion in non-human primates^5^,^6^,^7^. An alternative is to use live mice, which are versatile as a model, and accessible. The mouse aqueous drainage system closely resembles that of primates in structure and function, and mouse molecular tools or genetic strains with elevated IOP or outflow resistance are available^8^,^9^,^10^,^11^,^12^,^13^,^14^. To exploit the mouse for this purpose, it is necessary to stably perfuse the mouse anterior chamber, pharmacologically or molecularly probe the drainage tissue, and analyze physiology *in vivo*, but this is challenging to achieve by traditional methods used in larger mammals. To a large part this is because the mouse anterior chamber is so tiny with a volume of only about 5 μl^15^, making it technically challenging to achieve reliable measurement.

The extent to which aqueous drainage tissue actomyosin contractility modulates outflow resistance in mice is unknown. Mouse models represent powerful platforms on which to molecularly dissect poorly understood IOP regulatory mechanisms and treatment targets. Studies in the model are expected to inform on similarities between mouse and primate aqueous drainage systems, and applicability of mouse findings to humans. Establishing techniques to do so reliably should enhance our capacity to answer these questions.

We have dealt with the challenge of analyzing live mouse aqueous dynamics by appropriately scaling microperfusion techniques to the smaller mouse eye and coupling fluidic control to an electronic feedback system that yields stable and reproducible measurements *in vivo*^16^. Capacity to simultaneously deliver pharmacologic probes to the live mouse anterior chamber and drainage tissues and measure physiologic effect in real time has many potential applications. A further advantage of using live mice is the possibility that it is not plagued by the experimental ‘washout’ artifact of anterior chamber perfusion seen in larger non-human mammals, as mouse eyes *ex vivo* seem to be free of it^11^,^17^,^18^,^19^,^20^,^21^. The washout artifact describes a time-dependent decrease in outflow resistance of unclear etiology that confounds physiologic measurements. Whether live mice are free of this washout artifact has not been determined.

We used latrunculin-B (Lat-B) as a probe to analyze the *in vivo* effect of actomyosin inhibition on outflow resistance of the mouse drainage tissues^22^,^23^,^24^,^25^,^26^. A classical approach, 2-level alternating constant pressure perfusion^17^,^18^,^19^, was adapted and fine-tuned for mice. Outflow facility representing hydraulic conductivity; functions describing outflow resistance; presence of a washout artifact; and dose- and time-dependent drug responses were determined *in vivo*. The corresponding state of drainage tissue actin polymerization reflecting contractility in the mouse aqueous outflow system was then analyzed. Our findings are relevant to probing actomyosin outflow regulatory mechanisms, establishing structural-physiologic correlates, and developing novel glaucoma drugs in live mice as a representative model of the human system.

## Results

### Stable and reproducible physiologic measurement by 2-level constant pressure perfusion

**Figure 1:**
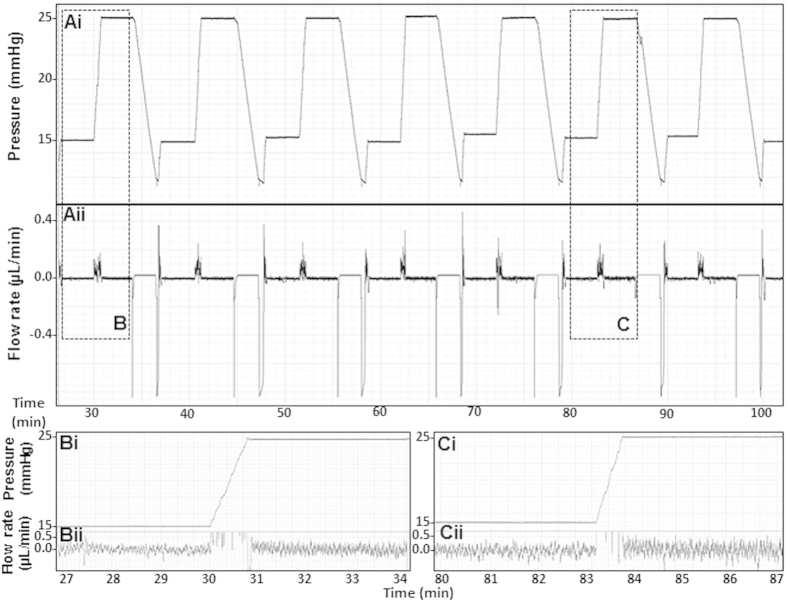
Example of pressure and flow rate traces for 2-level constant pressure perfusion. (**Ai**) pressure (y-axis; scale: 0.5 mmHg per square) vs. time (x-axis; scale 1 minute per square); (**Aii**) flow rate (y-axis; scale: 0.05 μL/min per square) vs. time (x-axis; scale 1 minute per square); (**B**,**C**) detail of pressure (i, y-axis; scale: 0.5 mmHg per square) and flow rate (ii, y-axis; scale: 0.1 μL/min per square) traces on a time scale (x-axis; scale 5 sec per square). Spikes in the flow trace reflect a momentary increase in flow rate as the pump speed increased to meet a new set-point pressure. Flow rate then automatically readjusted to a lower steady rate commensurate with the new set-point pressure.

Alternating 2-level constant pressure perfusions yielded stable and reproducible measurements, as shown in representative pressure and flow rate tracings in Fig. 1. This pattern was seen over perfusions lasting 2 hours, confirming our previous reproducibility studies over a shorter period^16^. Coefficients of variation for repeat measurements over 2-hour perfusions were under 2% for pressure (range 0.2–1.3%) and flow rate (range 0.3–1.7%).

### Absence of washout artifact in live mice

**Figure 2:**
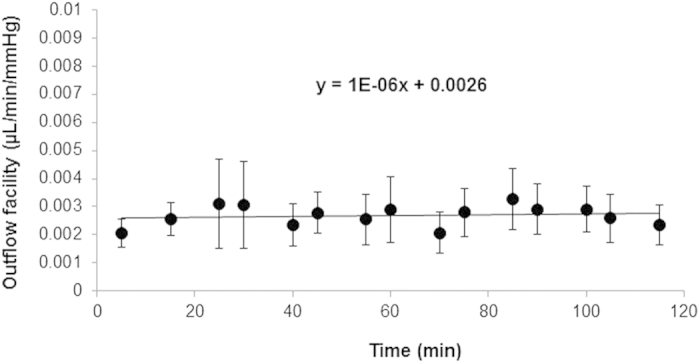
‘Washout’ artifact affecting outflow facility was not seen over 2-hour alternating 2-level constant pressure perfusions for 10 cycles of 15/25 mmHg in live mice. Regression slope of the outflow facility vs. time function was virtually flat (−0.000001; n = 7 mice).

Serial outflow facility (representing hydraulic conductivity) estimates during alternating 2-level constant pressure perfusion of Dulbecco’s phosphate buffered saline (DPBS) for 10 cycles each showed no significant facility change over 2 hours, as shown in Fig. 2 (17 total outflow facility estimates over 2 hours). Linear regression of serial outflow facility estimates over 2 hours showed a slope of zero (−0.000001; function: y = −0.000001x + 0.0026; n = 7 mice). A p-value of 0.76 for difference from zero slope indicated rejection of the null hypothesis that a time-dependent facility change occurred. A power calculation indicated that a sample size of n = 7 was sufficient to determine if perfusion over 120 minutes alone artificially elevated outflow facility (“washout effect”) by one and a third standard deviations; an increase of 0.00048 μL/min/mmHg or 17.8% above the mean of 0.0027 μL/min/mmHg (80% power; alpha = 0.05, one-tail). Thus live mouse outflow facility did not significantly increase (ie., ‘washout’ artifact absent) or decrease over 2-hour perfusions with DPBS (or 0.02% ethanol vehicle control). Mean total outflow facility for 2-level constant pressure perfusion over 2 hours was 0.0027 ± 0.00036 μL/min/mmHg.

### Lat-B dose- and time-dependently increased outflow facility in live mice

**Figure 3:**
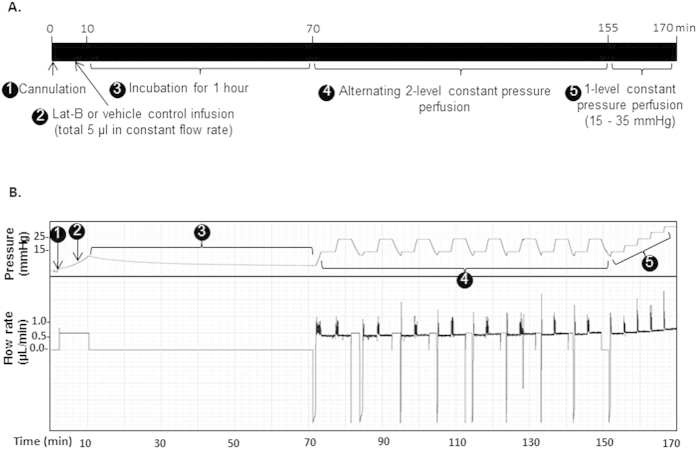
Schematic outline and representative perfusion pressure and flow rate traces following Lat-B anterior chamber delivery. (**A**) Timeline of perfusion studies, (**B**) Representative pressure and flow rate trace following 5 μM Lat-B delivery. 1: 35-gauge needle cannulation of live mouse anterior chamber ➊, 2: Lat-B or vehicle delivery at a constant flow rate ➋ (0.63 μL/min, total 5 μl); 3: 1-hour incubation ➌. 4: alternating 2-level constant pressure perfusion at 15 and 25 mmHg for 8 cycles ➍, followed by 5: step-wise, incremental constant pressure perfusion at incrementally elevated pressures from 15–35 mmHg ➎. Y-axis scale: 1 mmHg and 0.05 μL/min per square; x-axis scale: 2 minutes per square.

A protocol for Lat-B perfusion delivery to the live mouse anterior chamber is summarized in Fig. 3A. Briefly, Lat-B at different doses (2.5, 5, and 10 μM) was infused to a total volume of 5 μl into the anterior chamber of each eye by constant flow perfusion (0.63 μL/min). After anterior chamber Lat-B incubation for 1 hour, 2-level constant pressure perfusion was performed. Perfusion pressures were alternated between 15 and 25 mmHg for 8 cycles each. After this, the mouse eye was subjected to a step-wise, incremental elevation of constant pressure levels from 15, 20, 25, 30, to 35 mmHg, with each eye stably perfused at each pressure for at least 3 minutes, and corresponding flow rates were recorded, as previously described^16^. Vehicle control animals received ethanol 0.02% in DPBS and underwent outflow facility measurement according to the same protocol as animals receiving Lat-B. An example of perfusion pressure and flow tracings is shown in Fig. 3B.

**Figure 4:**
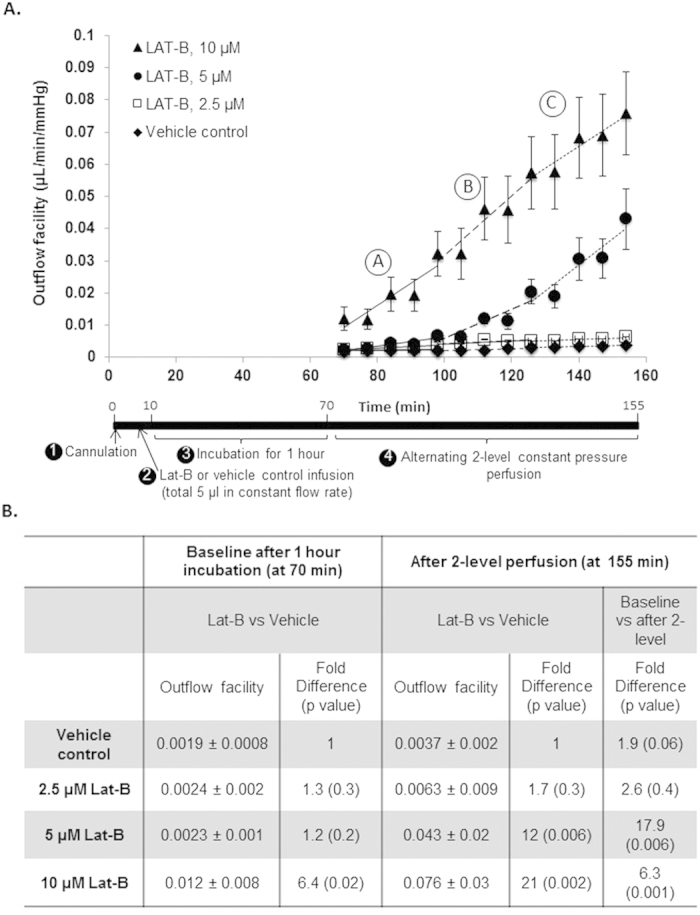
Lat-B dose-dependently increased outflow facility. (**A**) Lat-B (▲, 10 μM, n = 6; ●, 5 μM, n = 7; □, 2,5 μM, n = 5) or vehicle (♦, n = 5) was delivered to the live mouse anterior chamber and incubated for 1 hour, then 2-level constant pressure perfusion was performed. 5 μM and 10 μM Lat-B increased outflow facility compared with 2.5 μM and vehicle controls (ethanol 0.02%). Outflow facility following 2.5 μM Lat-B and vehicle was similar. Data points: mean outflow facility ± standard error of mean. (**B**) Statistical analysis of outflow facility based on 2-level constant pressure perfusion following live mouse anterior chamber Lat-B delivery.

Following delivery of 5 μM and 10 μM Lat-B, outflow facility significantly and time-dependently increased relative to baseline facility, which was determined from the first two outflow facility measurements following 1-hour drug incubation and ethanol 0.02% vehicle control facility, as shown in Fig. 4. A trend toward increased outflow facility was seen for 2.5 μM Lat-B relative to baseline and vehicle control facility, but this was not significantly different over the 2-hour perfusion period (p = 0.2). Onset of effect was observed earlier with higher doses, particularly for the 10 μM dose compared with 5 μM and 2.5 μM doses, as outflow facility with the former was already significantly higher right after drug incubation (p = 0.02).

Statistical analysis of outflow facility is summarized in Fig. 4B. Baseline outflow facility immediately after 1-hour incubation with 2.5 μM and 5 μM Lat-B was not significantly increased (p > 0.2) compared with vehicle control. Baseline facility following 10 μM Lat-B perfusion was 6.4 fold higher (p = 0.02) compared with vehicle controls.

The dose- and time-dependency of Lat-B effect was obvious at the end of 2-level constant pressure perfusion, when outflow facility was 18-fold (p = 0.006) and 6-fold (p = 0.001) higher compared with baseline for 5 μM and 10 μM Lat-B doses, respectively. At this late time-point (at 155 min), facility was significantly higher by 12-fold (0.043 ± 0.02 μL/min/mmHg, p = 0.006) and 21-fold (0.076 ± 0.03 μL/min/mmHg, p = 0.002) compared with vehicle controls for 5 μM and 10 μM Lat-B doses, respectively.

The time-dependent rate of outflow facility increase due to Lat-B was analyzed over 3 equal 30-minute perfusion periods, early (A; 70–100 min), middle (B; 100–130 min) and late (C; 130–160 min). The rate of facility increase (slope) for 10 μM Lat-B was highest throughout and at least an order of magnitude higher than the increase due to 2.5 μM Lat-B and vehicle controls. Compared with 10 μM Lat-B, the rate of 5 μM Lat-B-induced facility increase was 7 times lower in period A (1 × 10^−4^ (for 5 μM) vs. 7 × 10^−4^ (for 10 μM) μL/min/mmHg/min), but the rates were equivalent in period C (8 × 10^−4^ (for 5 μM) vs. 7 × 10^−4^ (for 10 μM) μL/min/mmHg/min). Compared with 2.5 μM Lat-B, the rate of 5 μM Lat-B-induced facility increase was double in period A (5 × 10^−5^ (for 5 μM) vs. 1 × 10^−4^ (for 2.5 μM) μL/min/mmHg/min) but an order of magnitude higher in the later B and C periods (5 × 10^−5^ (for 5 μM) vs. 5 × 10^−4^ (for 2.5 μM); and 4 × 10^−5^ (for 5 μM) vs. 8 × 10^−4^ (for 2.5 μM) μL/min/mmHg/min, respectively). Similar to control animals perfused with DPBS only, ethanol 0.02% vehicle control animals did not show a significant time-dependent facility change relative to baseline facility (p = 0.1), confirming the absence of ‘washout’.

### Flow rate and pressure relationship

**Figure 5:**
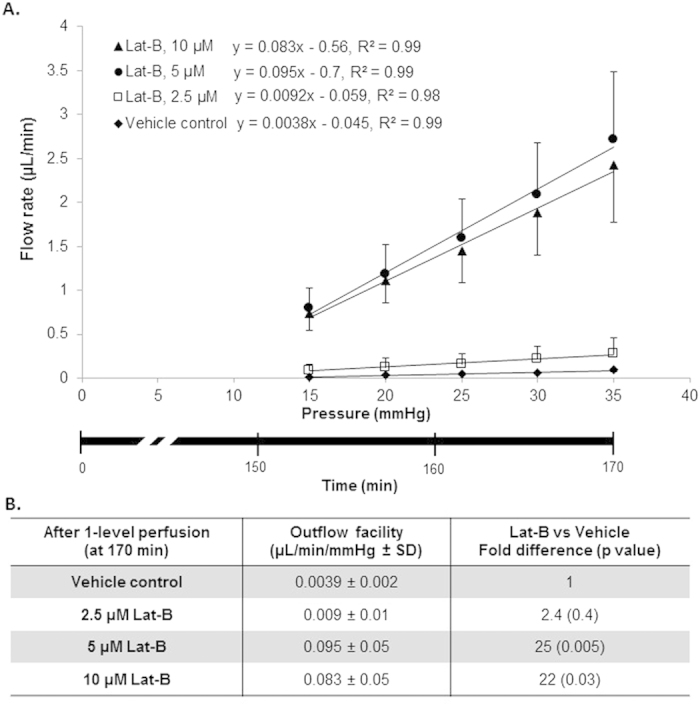
Lat-B dose-dependently increased outflow facility based on step-wise, incremental constant pressure elevation that followed 2-level constant pressure perfusion (step ➎ in Fig. 3). (**A**) The relationship between perfusion flow rate and pressure fit a linear function within a pressure range of 15–35 mmHg. Outflow facility following 5 μM (n = 7) and 10 μM (n = 6) Lat-B was higher compared with 2.5 μM (n = 5) Lat-B (p = 0.005) and vehicle controls (ethanol 0.02%, n = 5, p < 0.01). Outflow facility following 2.5 μM of Lat-B was not significantly different from vehicle controls (p = 0.4). Data points: mean ± standard error of mean. Lower x-axis: time following 2-level constant pressure perfusion. (**B**) Statistical analysis of outflow facility based on constant pressure perfusion at step-wise, incrementally elevated pressures between 15–35 mmHg after live mouse anterior chamber Lat-B delivery. SD, Standard deviation.

The step-wise constant pressure perfusion (155–170 min; 5 mmHg steps between 15–35 mmHg) that followed 2-level constant pressure perfusion (65–155 min) revealed a linear relationship between flow rate and perfusion pressure within a pressure range of 15–35 mmHg, as shown in Fig. 5. This observation was reproduced for Lat-B doses of 2.5, 5, and 10 μM. At this later time-point beyond the 2-level constant pressure perfusion period, outflow facility (slope) was similar for Lat-B doses of 10 μM and 5 μM (difference, p = 0.2), indicating possible convergence of facility-reducing effects of the different Lat-B doses over longer perfusions. Both doses still induced significantly higher facility (0.095 and 0.083 μL/min/mmHg for 5 μM and 10 μM Lat-B, respectively) compared with the 2.5 μM dose (0.009 μL/min/mmHg; about 10× higher, p = 0.005) and vehicle controls (0.0039 μL/min/mmHg; about 20× higher, p = 0.03). Outflow facility after 5 μM and 10 μM Lat-B was 25-fold (p = 0.005) and 22-fold (p = 0.03) higher respectively compared with vehicle controls. Outflow facility following Lat-B 2.5 μM was not significantly different from vehicle controls (p = 0.4).

### Time-dependent pattern of altered outflow resistance

**Figure 6:**
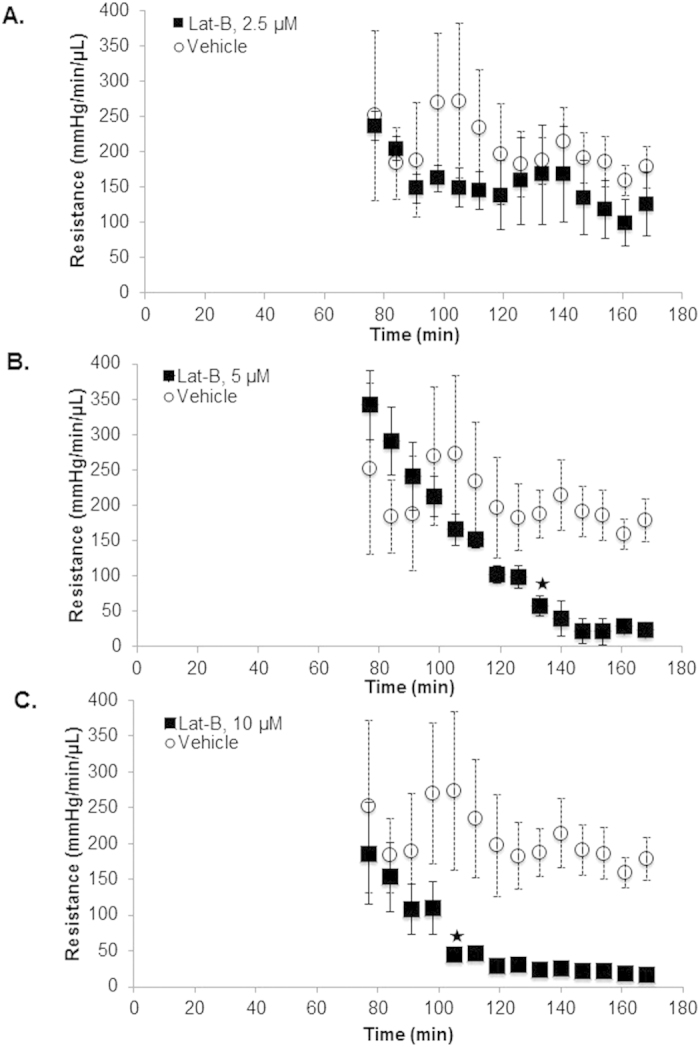
Outflow resistance functions following Lat-B exposure in live C57BL/6 mice. Data points represent mean resistance (error bars: standard error of mean; mmHg/min/μL) following different doses of Lat-B ((**A**) 2.5 μM, n = 5; (**B**) 5 μM, n = 7; (**C**) 10 μM, n = 6) compared with vehicle (0.02% ethanol, n = 5). Star: time-point at which outflow resistance became significantly lower compared with vehicle controls (both p < 0.01).

Outflow resistance following Lat-B anterior chamber delivery and 2-level constant pressure perfusion is shown in Fig. 6. 5 μM and 10 μM Lat-B significantly time- and dose-dependently reduced outflow resistance compared with vehicle controls. Reduced outflow resistance due to 10 μM Lat-B reached statistical significance 30 minutes before 5 μM Lat-B (Fig. 6B,C; star; p < 0.01), although the resistance was reduced to similar levels over time with both doses. A trend toward reduced outflow resistance relative to vehicle controls was seen following 2.5 μM Lat-B, but this did not reach statistical significance over the period of perfusion (p = 0.2).

### Lat-B effect on aqueous drainage tissue actin polymerization

**Figure 7:**
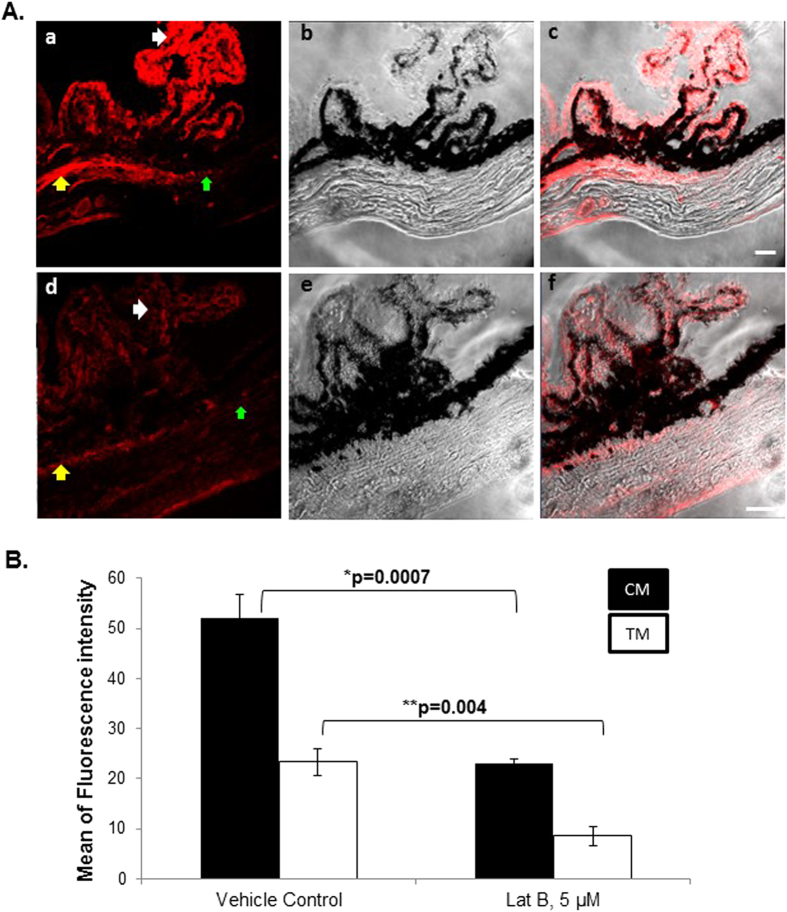
Live mouse anterior chamber Lat-B delivery decreased F-actin fluorescence labeling in aqueous drainage tissues. (**A**) F-actin in vehicle controls (ethanol 0.02%, (a)), phase contrast image (b), merged F-actin labeling with phase contrast image (c), F-actin labeling following exposure to Lat-B (5 μM, (d)). Phase contrast image (e), F-actin labeling merged with phase contrast image (f). Yellow arrow: ciliary muscle (CM); green arrow: trabecular meshwork (TM); white arrow: ciliary process. Scale bar, 20 μm. (**B**) Quantitative analysis of F-actin fluorescence labeling in aqueous drainage tissues after Lat-B anterior chamber delivery in live mice. Fluorescence intensity (mean ± standard error of mean) was measured as grey pixel values in 8 regions of interest within TM or CM (n = 4 mice per label) following Lat-B (5 μM) or vehicle (ethanol 0.02%) exposure. In both CM and TM, F-actin fluorescence intensity was significantly lower following Lat-B exposure (p < 0.005).

Polymerized actin (filamentous actin; F-actin) served as an indicator of contractile state. In vehicle control tissue, the density and distribution of labeled F-actin was prominent in the ciliary muscle (yellow arrow) and ciliary body (white arrow), and somewhat less in the trabecular meshwork (TM, green arrow), representing a basal state of contractile tone as shown in Fig. 7A(a–c). The distribution and intensity of F-actin labeling in vehicle control tissue was similar to that of eyes perfused with DPBS only (data not shown). Anterior chamber perfusion of 2.5, 5 and 10 μM Lat-B caused significantly reduced F-actin fluorescence labeling intensity in the drainage structures of ciliary muscle (p = 0.0007) and TM (p = 0.004), as shown in Fig. 7A(d–f) for 5 μM Lat-B as a representative finding. F-actin labeling intensity was not different between different Lat-B doses of 2.5, 5 and 10 μM (data not shown).

Following 5 μM Lat-B anterior chamber perfusion, mean fluorescence intensity of F-actin labeling in ciliary muscle and TM was decreased by 48% and 46%, respectively (both p < 0.005) compared with vehicle controls in quantitative immunohistochemistry image analysis, as shown in Fig. 7B.

**Figure 8:**
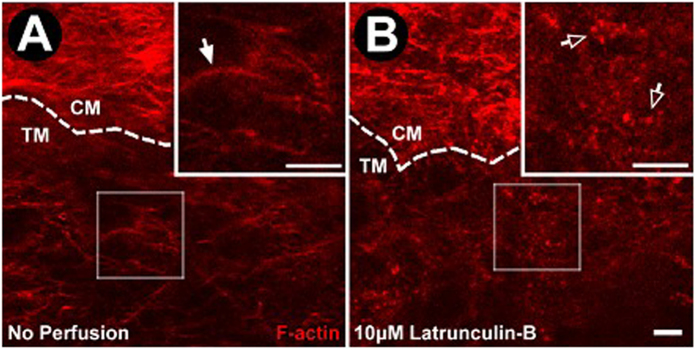
Effect of Lat-B on aqueous drainage tissue F-actin following live mouse drug delivery. (**A**) F-actin labeling (red) of unperfused control eye (n = 3 mice). Ciliary muscle (CM) F-actin labeling is brighter and denser compared with adjacent trabecular meshwork (TM). Cortical F-actin organized as a curvilinear network is prevalent without punctate F-actin. (**B**) F-actin after Lat-B (10 μM, n = 3) delivery in live mice. The curvilinear F-actin network was absent but punctate F-actin was prominent. Closed arrows: Cortical F-actin organized as curvilinear network. Open arrows: Punctate F-actin. Dashed lines: CM-TM border. Insets: 2× magnification of sample region in the TM. Semi-transparent boxes: indicate region of TM selected for magnification in inset. Scale bar, 25 μm.

High resolution *in situ* transscleral 2-photon excitation fluorescence imaging (TPEF) further confirmed the effect of Lat-B on F-actin in the mouse aqueous drainage tissues, as shown in Fig. 8. In control mice, a curvilinear network of F-actin that was denser and more intense in the ciliary muscle than in the adjacent TM was seen. Following exposure to 10 μM Lat-B as a representative dose, F-actin labeling that was initially arranged as a curvilinear network rearranged as aggregates.

## Discussion

Our system incorporating electronically controlled microperfusion fluidics coupled to real-time pressure feedback permitted stable and reproducible 2-level constant pressure perfusion over prolonged periods in live mice. The washout artifact that characterizes many large mammal perfusions was not seen in live mice, with either DPBS or 0.02% ethanol vehicle. A dose- and time-dependent increase in total outflow facility representing a graded increase in total outflow facility was seen in response to Lat-B-induced inhibition of actomyosin contractility. Our facility measurement system was sensitive enough to detect and quantify this change. Lat-B exposure was associated with reduced actin polymerization in the mouse drainage tissues of TM and ciliary muscle. These findings reflect the importance of actomyosin contractility to aqueous outflow modulation in live mice, mirroring functional aspects of the primate drainage system.

Outflow facility values may vary according to perfusion technique, calculation method (eg., weighted successive averages; linear regression)^27^, and tissue state (*in vivo*^9^,^10^,^12^,^28^,^29^,^30^ or *ex vivo* tissue^11^,^13^,^31^,^32^). In mice, a broad facility range of 0.005–0.039 μL/min/mmHg has been reported. This range may be segregated according to experimental conditions: typically, *in vivo* C57BL/6 mouse mean outflow facility values derived by constant flow perfusion (0.015–0.025 μL/min/mmHg)^12^,^28^,^33^ are higher than values derived by constant pressure perfusion (0.005–0.007 μL/min/mmHg^9^,^10^,^16^,^29^,^30^). Our own reported *in vivo* C57BL/6 mouse mean facility values derived by constant pressure perfusion (0.0066 μL/min/mmHg^16^) were in this 0.005–0.007 μL/min/mmHg range. The higher C57BL/6 mouse *in vivo* values obtained by constant flow perfusion (0.015–0.025 μL/min/mmHg) are approximated only by measurements performed *ex vivo* (0.0093–0.029 μL/min/mmHg)^13^,^31^,^32^,^34^.

We chose to perform two-level constant pressure perfusion at alternating pressures that were lower than used by other groups (15/25 mmHg vs. 25/35 mmHg)^9^,^10^. Higher pressures used in other mouse studies mimic those used in classic primate perfusion studies^5^,^23^,^25^,^26^, but we chose the lower pressures to be physiologically relevant to the C57BL/6 mouse IOP (11–16 mmHg)^35^,^36^. Additionally, we had found less flow rate instability at these lower pressures^16^.

Our previous report of *in vivo* mean outflow facility (0.0066 μL/min/mmHg) was based on linear regression of flow rate data obtained at multiple pressures between 15–35 mmHg^16^. While our previous data^16^ closely fit a linear regression model (y = 0.0066x − 0.034; R^2^ = 0.97), a reanalysis showed that the flow vs. pressure function was actually flatter at lower pressures (15–25 mmHg; slope: 0.003 ± 0.0015 μL/min/mmHg) than at higher pressures (25–35 mmHg; slope: 0.008 ± 0.005 μL/min/mmHg). In fact, recalculation of facility at lower perfusion pressures (mean 0.003 μL/min/mmHg at 15–25 mmHg) closely agreed with our present facility observation (mean 0.0027; range 0.002–0.0039 μL/min/mmHg) determined at similar pressures (15/25 mmHg; p = 0.2; n = 8 mice per group).

Thus using lower perfusion pressures *in vivo* appears to explain the lower facility findings reported here, as compared with previously reported facility values derived by higher perfusion pressures. Based on our and foregoing reports, we conclude that facility findings may vary according to (1) differences in methodology (constant pressure vs. constant flow) and tissue state (live animal vs. postmortem tissue), but also (2) choice of perfusion pressure.

Lat-B dose- and time-dependently increased total outflow facility in live mice. Facility increased by up to 12- and 21-fold for 5 μM and 10 μM Lat-B respectively compared with vehicle controls. While 2.5 μM Lat-B did not significantly increase facility over that of vehicle controls over the perfusion timeframe, a trend toward gradually increased facility over time was seen that perhaps was not captured within the duration of our perfusions. The onset of action of 10 μM Lat-B was quicker than that of 5 μM, and the onset for 5 μM was quicker than that of 2.5 μM Lat-B. Facility at later time-points for the 5 μM and 10 μM doses was similar, however. Resistance analysis reflected this too. This similarity suggests that while 10 μM and 5 μM Lat-B doses induced different physiologic effects earlier in the perfusions, their effects converged over time. This indicates that a Lat-B dose of about 5 μM optimally reduced outflow resistance in live mice.

Lat-B delivery in live mice was performed by constant flow perfusion to a volume of 5 μl at each dose. The 5 microliter delivery volume, which approximates the anterior chamber volume of mice, was standardized over all dosages to allow for comparison of dose-dependent effect. Standardized perfusion duration and volume allowed valid comparisons of the different drug doses. Drug perfusion flow rates were chosen to maintain physiologically relevant conditions, with transduced perfusion pressure being kept in the range of 15–20 mmHg for the roughly 8-minute drug delivery perfusions. We are presently validating a promising method^37^ to exchange anterior chamber contents and correctly estimate drug dose in the anterior chamber that could be used in the future to achieve a desired anterior chamber drug concentration.

The facility responses to Lat-B we observed may not be directly comparable to the latrunculin dose-responsiveness reported in live non-human primates^24^,^27^,^38^,^39^ as we did not directly exchange the anterior chamber contents. For example, while a 4–5 fold facility increase following 2 μM of Lat-B was seen 90 min after exchange perfusion in monkeys, we did not find a significant increase in facility for the same dose over the same period in mice. The exact anterior chamber drug concentration following initial drug delivery was unknown in the *in vivo* mouse model, but would have been lower than the dose delivered due to anterior chamber drug dilution and drug escape through the drainage pathway. Anterior chamber drug concentration would have increased with ongoing drug perfusion, however, and likely was reflected in the significant facility increase seen over time with our perfusions of 5 and 10 μM Lat-B.

Absence of the washout artifact in live C57BL/6 mice confirms *ex vivo* studies in enucleated eyes of the same mouse strain^11^. Thus unlike studies in larger mammals, perfusion data obtained in live mice do not need to be statistically corrected or adjusted for the confounding washout artifact over the perfusion duration we used. The washout artifact is not seen in human eyes either^17^, further indicating the attractiveness and relevance of mice for modeling human outflow dynamics *in vivo*.

A conceptual difference between traditional techniques of perfusion under constant hydrostatic pressure and our electronically controlled microperfusion system is that our system delivers nano to microliter-scale pulses of fluid to maintain a constant pressure in the mouse anterior chamber. The pump perfusion is intermittent, activating only when pressure dips below a preset threshold to restore the constant pressure. Conversely, no perfusion occurs while pressure remains above the threshold. By contrast, traditional perfusion systems subject the anterior chamber to the constant pressure of a hydrostatic column as long as the tubing is open. Whether our constant pressure perfusion methodology contributed to the stable and reproducible measurements and lack of washout artifact that we observed remains to be determined.

Lat-B sequesters monomeric actin to prevent polymerization and formation of actin filaments, the contractile form of actin^38^,^39^,^40^. Not surprisingly, Lat-B delivery to the anterior chamber was associated with decreased actin polymerization in the aqueous drainage tissues. This was seen as significantly reduced F-actin labeling intensity and rearrangement of the curvilinear F-actin network in the mouse drainage tissues. The finding that reduced actin polymerization in the drainage tissue correlated with increased outflow facility following Lat-B exposure agreed with reports in non-human primates^23^,^24^. These findings indicate that actomyosin mechanisms modulating outflow facility are common to primates and mice.

We compared the effects of different concentrations of Lat-B (2.5, 5, and 10 μM) on F-actin fluorescence. We found a significant difference in F-actin intensity/distribution between Lat-B treatment and vehicle, but we did not find F-actin differences between the different Lat-B dosages (data not shown). Subtle differences in actomyosin contractility corresponding to differences in Lat-B dose-related facility change may not have been evident by F-actin analysis alone, or perhaps our techniques could not resolve them.

While we expected to find decreased actin polymerization in the TM following Lat-B perfusion, we did not expect to see this in ciliary muscle. Perhaps this finding is not surprising, however, given that the ciliary muscle forms the anterior part of the uveoscleral drainage pathway exposed to aqueous humor contents as aqueous exits the anterior chamber. Ciliary muscle extensions also intermingle with the TM and affect conventional outflow facility. Ciliary muscle is smooth muscle^41^ that is subject to actin polymerization. Primate uveoscleral outflow is considered to be only minimally pressure sensitive under conditions where IOP is less than episcleral venous pressure^42^. It is possible, however, that the uveoscleral pathway becomes more pressure sensitive under the influence of factors such as intraocular inflammation and prostaglandins^43^,^44^,^45^,^46^,^47^,^48^. Relaxation of the ciliary muscle itself mediates increased uveoscleral outflow^49^,^50^,^51^,^52^, but whether this also alters the pressure sensitivity of the pathway is unclear. To what extent outflow resistance is influenced by relative contributions and crosstalk between different contractile and extracellular matrix elements in different regions of a complex drainage pathway is still being worked out^53^. To our best knowledge, increased outflow facility by latrunculin or other actomyosin-altering agents has not previously been attributed to altered ciliary muscle actomyosin contractility^54^. Our findings in live mice suggest that such a mechanism is worth exploring further.

## Methods

### Animal Husbandry and Anesthesia

Mouse experiments were performed in accordance with the ARVO Statement for Use of Animals in Ophthalmic and Vision Research. C57BL/6 mice aged 3–4 months were purchased from Charles River Laboratories (Wilmington, MA). Approval was obtained from the University of Southern California Institutional Animal Care and Use Committee (IACUC). The mice were raised and housed in air-filtered clear cages with a bedding of pine shavings, subject to a 12-hour light/dark cycle, and fed *ad libitum*. All perfusion measurements were performed between 12 pm and 5 pm.

Mice were anesthetized with a mixture of ketamine (60–85 mg/Kg, Ketaject, Phoenix Pharmaceutical, Inc., St. Joseph, MO), xylazine (6–8.5 mg/Kg, AnaSed; Lloyd Laboraties, Shenandoah, IA) and acepromazine (1.5–2.5 mg/Kg, Boehringer Ingelheim, St. Joseph, MO), injected intraperitoneally. Anesthesia was titrated to achieve a depth facilitating stable anterior chamber cannulation and perfusion. One drop of topical proparacaine hydrochloride ophthalmic solution (0.5%, Akorn, Inc, Buffalo Grove, IL) was applied to the cornea prior to needle cannulation. Mice were rested on a warming platform or under a heating blanket to maintain body temperature during experiments.

### Two-level Constant Pressure Perfusion

Anterior chamber perfusion was performed using previously described apparatus and methods^16^. With our two-level constant-pressure perfusion in live C57BL/6 mice (n = 7 mice), the perfusion pressure set-point was alternated between 15 mmHg and 25 mmHg. The pump had infusion and withdrawal modes that allowed perfusion pressure to be increased from 15 to 25 mmHg, decreased from 25 to 15 mmHg, then increased again to 25 mmHg and so on, alternating between 15 and 25 mmHg. Typically, perfusion at each pressure started with a 30-second equilibration period followed by perfusion for 3 minutes at that pressure. Each perfusion cycle comprised data collected during perfusion at 15 then 25 mmHg. Alternating 15/25 mmHg perfusion was repeated 8–10 times. This yielded 8–10 sequential 15/25 mmHg perfusion cycle data sets representing total data from 16–20 3-minute perfusion periods per eye (performed 10 sets for Figs 2 and 8 sets for Fig. 4). One eye per animal was perfused. Outflow facility (C) was calculated by the standard method of weighted successive averages, as classically performed in primate perfusions^17^,^19^. Briefly, outflow facility (C) following Lat-B incubation was calculated from data sets of pressure (P1 − P16) and corresponding flow rates (F1 − F16), as illustrated in Supplementary Fig. 1. For successive 15/25 mmHg perfusion cycles, C1 through C15 was calculated by equation (1).


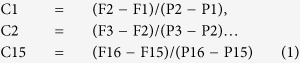


The first total outflow facility value (Ct^1^) was calculated as the average of C from the first three successive perfusion cycles (C1 − C3). To calculate the next Ct value (Ct^2^), the oldest data point from the first perfusion cycle (C1) was excluded and the subsequent C value (C4) added to the prior two overlapping periods (C2 and C3), as described by equation (2).


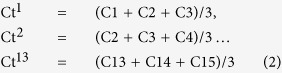


Outflow resistance was calculated as the inverse of facility measurements, having units of mmHg/min/μL. Following this method of two-level constant-pressure perfusion, total outflow facility was further determined in the same live mice by constant pressure perfusion for at least 3 minutes at (“step-wise”) incrementally higher pressure levels between 15–35 mmHg (15, 20, 25, 30 and 35 mmHg), with total outflow facility given by the regression slope of the flow rate (F; μL/min) vs. pressure (P; mmHg) function.

To determine total outflow facility in 2- level or step-wise perfusion, pressure and flow rate data were extracted from the software at a rate of 1 per 10 milliseconds for 15,000 consecutive data points for each pressure condition. Data from the first 30 seconds (3000 data points) were taken to occur during system stabilization and excluded. The pressure transducer and pump were calibrated as previously described^16^. Statistical analysis was performed in Excel^®^ 2008 for Mac (Microsoft, Redmond, WA), StatPlusMac 5.7.5 (AnalystSoft Inc., Alexandria, VA) and Minitab^®^ 9.2 for Windows (Minitab Inc., State College, PA).

### Drug Delivery of Latrunculin-B

Latrunculin-B (Lat-B; EMD Millipore, Billerica, MA) was prepared in 100% ethanol (EMD) as a 25 mM stock solution. Lat-B solution for perfusion was freshly prepared in Dulbecco’s phosphate buffered saline (DPBS; Mediatech, Corning) with final concentration of 0.02% ethanol. Perfusion apparatus was filled and primed with Lat-B of different concentrations (2.5, 5, and 10 μM; n = 5, 7, and 6 mice for each dose, respectively) for perfusion at the respective concentrations.

Drug delivery of Lat-B into the anterior chamber of live C57BL/6 mice was performed at a constant flow perfusion rate of 0.63 μL/min for 7–8 minutes approximately, depending on pump calibration, for a total of 5 μl volume delivered. This allowed for standardized volume delivery of drug at the different concentrations into the anterior chamber of all mice. During perfusion drug delivery, transduced anterior chamber pressure did not exceed 25 mmHg. After drug delivery was completed, perfusion was stopped and drug left to incubate in the anterior chamber for 1 hour. After drug incubation, the same apparatus was used to conduct alternating 2-level constant pressure perfusion (at 15 and 25 mmHg) as described above. This was then followed by step-wise, incremental constant pressure elevation (15–35 mmHg). Independent mice received ethanol 0.02% (vehicle control, n = 5 mice) or DPBS (non-vehicle control; n = 5 mice) in the same manner as mice receiving drug (Lat-B).

### Fluorescence Microscopy and Quantitative Analysis

After perfusion, enucleated eyes were quickly embedded in Tissue-Tek Optimum Cutting Temperature compound. Cryosections (7 μm thickness) were fixed with 4% paraformaldehyde (PFA) and further permeablized/blocked in the blocking solution (5% Bovine serum albumin and 0.3% Triton X-100) for 1 hour at room temperature (RT). To visualize the effect of Lat-B on filamentous actin (F-actin), the contractile form of actin, sections were incubated with Alexa 568-phalloidin (Life Technologies, Grand Island, NY) for 1 hour at RT and then mounted using ProLong Gold Anti-fade reagent with 4′,6-diamidino-2-phenylindole (DAPI, Life Technologies). Negative, non-specific labeling was established with normal IgG isotypes. Sections were analyzed with a Leica SP5 high-speed spectral confocal laser-scanning microscope (Leica Microsystems, Wetzlar, Germany) or a Zeiss LSM 710 confocal microscope (Carl Zeiss, Oberkochen, Germany). Immunofluorescence staining for phalloidin staining was performed in randomly selected slides (4–5 slides per each eye, n = 4 mice) containing 4 sections per slide and examined under the confocal microscope.

Specific fluorescence from tissue labeling in histological sections was captured by confocal microscopy with exposure time kept constant across all images. Image sections were imported as 16 bit images and analyzed by NIH Image J software. Fluorescence intensity of pixel grey values in 8 separate regions of interest per region of TM and CM was calculated and averaged across each tissue region. Fluorescence intensity for F-actin was measured in TM and CM separately, averaged across data from 4 mice, and then compared using ANOVA and Tukey’s and Sidak’s comparison tests^38^.

Additional frozen sections were stained with hematoxylin and eosin. Drainage tissue integrity and effect of Lat-B on tissue histological features of mouse eye after perfusion were examined by light microscopy (data not shown).

### Two-photon excitation fluorescence imaging

*Ex vivo* mouse eyes were fixed with 4% PFA and labeled intact, without dissection or sectioning, with Alexa 568-conjugated phalloidin (filamentous actin) and imaged by TPEF (Zeiss 710NLO [Oberkochen, Germany]^55^,^56^ supplemented with a BiG non-descanned detector [Zeiss]) to capture red fluorescence and second harmonic generation (SHG) images. SHG of mouse iridocorneal angle (not shown) was used to identify the region of the TM just deep to Schlemm’s canal by a transscleral approach^57^,^58^. In mice, the ciliary muscle lies just deep to the TM^15^. The eyes of non-pigmented Balb/c mice permitted unimpeded views of the deeper aqueous drainage tissues, which we have characterized previously^41^. Mice that were exposed and unexposed to 10 μM Lat-B (n = 3 mice) were imaged.

## Additional Information

**How to cite this article**: Ko, M.H. K. *et al.* Dose- and time-dependent effects of actomyosin inhibition on live mouse outflow resistance and aqueous drainage tissues. *Sci. Rep.* **6**, 21492; doi: 10.1038/srep21492 (2016).

## Supplementary Material

Supplementary Information
