# Supplementary material for: Dose- and time-dependent effects of actomyosin inhibition on live mouse outflow resistance and aqueous drainage tissues
Source: Sci Rep. 2016 Feb 17;6:21492. doi: 10.1038/srep21492 (PMC4756686; doi:10.1038/srep21492)
Supplement: Supplementary Information [file srep21492-s1.doc]

**Title:**

**Dose- and time-dependent effects of actomyosin inhibition on live mouse outflow resistance and aqueous drainage tissues**

**Authors:**

MinHee K. Ko, PhD

Eun Kyoung Kim, MD

Jose M. Gonzalez Jr, PhD

James C. Tan, MD, PhD

**Supplementary Figure 1:**


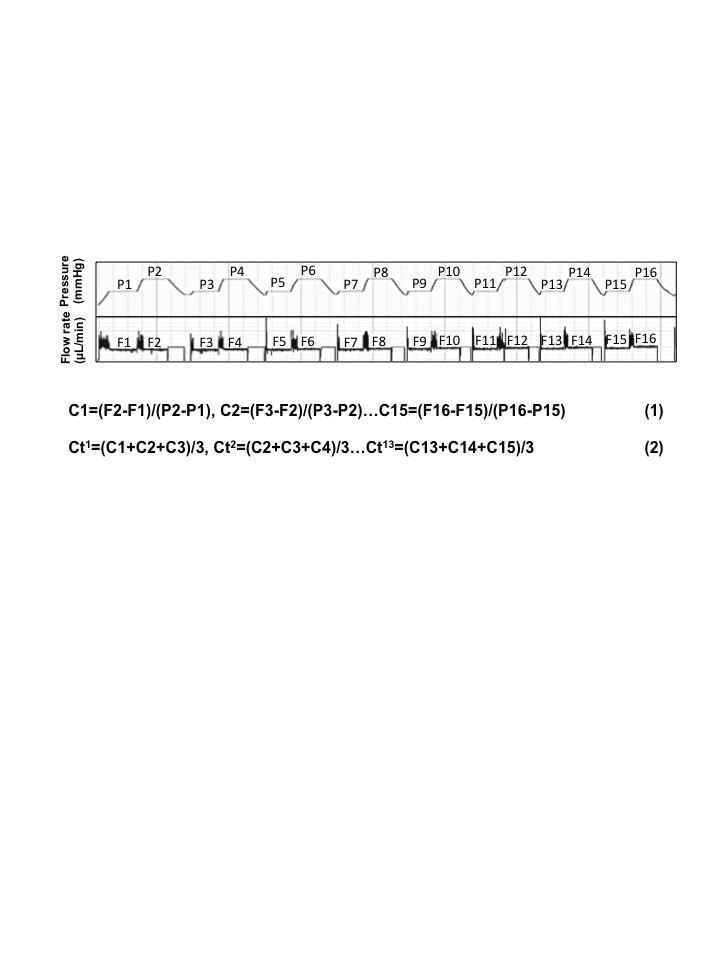


Supplementary Figure 1. Calculation of outflow facility from alternating two-level constant pressure perfusion. Outflow facility (C) was calculated from data sets of pressure (P1-P16) and corresponding flow rates (F1-F16). For successive 15/25mmHg perfusion cycles, C1 through C15 was calculated by equation (1). The first total outflow facility value (Ct^1^) was calculated as the average of C from the first three successive perfusion cycles (C1-C3). Calculate of the next Ct value (Ct^2^) and so on is described by equation (2).
